# Functional range of motion for basic seated activities of daily living tasks

**DOI:** 10.3389/fspor.2025.1646326

**Published:** 2025-08-29

**Authors:** Yuji Inagaki, Tomoya Ishida, Hiroyuki Sugimori, Takaaki Yoshimura, Akihiro Watanabe, Yumene Naito, Daisuke Sawamura

**Affiliations:** ^1^ Department of Rehabilitation Science, Faculty of Health Sciences, Hokkaido University, Sapporo, Japan; ^2^ Department of Biomedical Science and Engineering, Faculty of Health Sciences, Hokkaido University, Sapporo, Japan; ^3^ Department of Health Sciences and Technology, Faculty of Health Sciences, Hokkaido University, Sapporo, Japan; ^4^ Graduate School of Health Sciences, Hokkaido University, Sapporo, Japan; ^5^ Graduate School of Health Sciences, Kobe University, Kobe, Japan; ^6^ Department of Physiotherapy, Faculty of Medicine, Dentistry and Health Sciences, University of Melbourne, Melbourne, VIC, Australia

**Keywords:** kinematics, activities of daily living, eating, drinking, washing, functional limitation, rehabilitation

## Abstract

**Introduction:**

Efficient performance of activities of daily living (ADLs) requires coordinated movement across multiple upper-limb joints. However, current assessments of joint range of motion (ROM) during ADLs often rely on subjective evaluation and lack precise quantitative data. The functional ROM required for upper-limb movements in a seated position remains unclear, despite its clinical relevance for older adults and individuals with mobility limitations who frequently perform ADLs while seated. Additionally, little is known about how joint-motion requirements differ across similar ADL tasks, such as eating with a spoon versus chopsticks or washing the top versus the back of the head. To address these issues, we aimed to establish standardized ROM values for common upper-limb–related ADLs using three-dimensional motion analysis to enhance rehabilitation goal setting.

**Methods:**

Thirty-one healthy adults (14 women; mean age 22.9 ± 1.9 years) completed six seated ADLs—face washing; hair washing (top, back); chopstick or spoon eating; bottled-water drinking. Marker-based motion capture (International society of biomechanics guidelines) recorded kinematics. Descriptive statistics and paired t-tests (*p* < 0.05) assessed task differences.

**Results:**

Significant differences in upper limb and neck joint angles were observed across ADL tasks. Shoulder elevation was highest during back hair washing (105.0° ± 14.6°) and lowest when eating with chopsticks (39.2° ± 10.9°). Elbow flexion peaked during face washing (122.3° ± 5.2°) and back hair washing (127.9° ± 5.7°), reflecting the need for close hand-to-face contact. Wrist extension was greatest during face washing (−28.7° ± 8.5°), while a significant difference was found between chopstick (−13.7° ± 12.5°) and spoon use (−5.6° ± 5.3°, *p* = 0.005), indicating task-specific hand control demands. Neck flexion also varied significantly between hair washing conditions (back > top, *p* < 0.001). Furthermore, when eating with a bowl rather than with a plate, participants showed significantly greater shoulder elevation, elbow flexion, and forearm rotation (*p* < 0.01), suggesting increased ROM demands shaped by Japanese eating customs.

**Discussion:**

These reference ROMs offer objective targets for seated-ADL rehabilitation and assistive-device design. validation in older adults and clinical populations is warranted to confirm applicability and guide goal setting.

## 1 Introduction

Activities of daily living (ADLs), such as eating and hair washing, require coordinated movements of multiple upper limb joints, including the shoulder, elbow, and wrist (1, 2). A limited range of motion (ROM) makes it difficult for an individual to perform basic activities independently, such as eating and dressing, which directly affects quality of life. Therefore, objectively clarifying and quantifying the ROM required for specific ADL tasks is extremely important in rehabilitation. To maximize the effectiveness of upper limb rehabilitation, the therapist must accurately assess the ROM required for each ADL and set that value as a specific goal for functional recovery (3, 4).

Standardized assessments of ADL, such as the Barthel Index, Functional Independence Measure, and Modified Katz ADL Scale, which are widely used today, provide scoring based on observation and self-report, and the scores are influenced by the subjectivity and response bias of the evaluator (5, 6). In addition, since these are self-reliance rating scales based on observation, they are not as sensitive in detecting change. Therefore, it is necessary to establish quantitative indicators (objective measures) to serve as markers for evaluating and treating ADL.

Recent advances in motion sensor technology and three-dimensional (3D) motion analysis have enabled objective measurements of complex ADL movements (7–11). The ROM of the major joints in healthy individuals has been reported using electromagnetic sensors, motion capture with optical markers, force sensors, and inertial measurement units (2, 7, 12–15). However, quantitative indicators that comprehensively describe the ROM of the upper limbs and trunk during each ADL remain insufficiently established, posing issues for their use as objective markers in ADL evaluation and treatment (16). Furthermore, the way ADLs are performed is influenced by social and individual factors, such as cultural background, gender, and age (17–19). Consequently, van Andel et al. indicated that selecting representative ADL movements and the lack of functional ROM data remain challenging, as protocols and reference values for comprehensive movement analysis of the entire upper limb have not yet been standardized (16). In addition, there is a need to expand data collection for ADL tasks owing to the insufficient definition of motor mobility and the high degree of task dependence (20, 21).

In this study, we focused on two categories of ADLs identified in the Barthel Index—eating and grooming—that can be performed in a seated position. Specifically, we selected four representative ADL tasks: face washing, hair washing, eating, and drinking. Dressing movements were excluded owing to technical limitations associated with 3D motion capture; reflective markers are often occluded by clothing during such tasks, making accurate kinematic analysis difficult (22). Importantly, in contrast to those in Western countries, it is common practice in Japan to perform face and hair washing while seated, reflecting cultural habits (23). However, existing motion analysis studies rarely specify postural conditions and often assume standing positions by default (7, 24, 25). Moreover, there is a lack of comparative data for different components of the hair-washing task, particularly between movements directed toward the top and back of the head. While some studies have examined differences between chopstick and spoon use, few have captured simultaneous trunk and neck motion, which limits comprehensive evaluation of upper-body coordination during eating (17). Additionally, most previous studies have used cups to evaluate drinking tasks, whereas we adopted the use of a polyethylene terephthalate (PET) bottle to more closely replicate common real-life scenarios and to introduce novelty.

Based on these considerations, we selected four seated ADL tasks—face washing, hair washing, eating, and drinking—for detailed analysis. Furthermore, we subdivided hair washing into top-of-the-head and back-of-the-head movements and eating into chopstick-with-plate and spoon-with-bowl conditions, resulting in six total ADL task variations analyzed in this study. Using a 3D motion capture system, the joint angles of the shoulder, elbow, forearm, hand, trunk, and neck were analyzed, and the reference functional ROM required for basic ADL was provided. Through this analysis, we aimed to provide quantitative knowledge that contributes to the standardization of upper limb ADL movement analysis and rehabilitation goal setting.

## 2 Materials and methods

### 2.1 Participants

In this study, we included 31 healthy young adults (14 female and 17 male participants; mean age: 22.9 ± 1.9 years; height: 170.6 ± 6.8 cm; body weight: 61.8 ± 5.4 kg). The required sample size for this study was determined with reference to a previous study (26), using G*Power (version 3.1.9.7) (27). The calculation was based on a statistical power of 0.90 and a significance level of *α* = 0.05, yielding a required sample size of 28. To account for a potential 10% dropout rate, we recruited a total of 31 participants. All participants self-reported as right-handed. Recruitment was conducted via posters displayed on university bulletin boards. Participants were excluded if they reported pain or difficulty during any of the ADLs, including hair and face washing, eating with chopsticks or spoons, or drinking bottled water. Each participant provided written informed consent before participation. This study was approved by the Ethics Committee of the Faculty of Health Sciences at Hokkaido University (approval number: 24-35) and was conducted in accordance with the Declaration of Helsinki and Consolidated Standards of Reporting Trials guidelines.

### 2.2 Procedures

#### 2.2.1 Activity of daily living tasks

**Figure 1:**
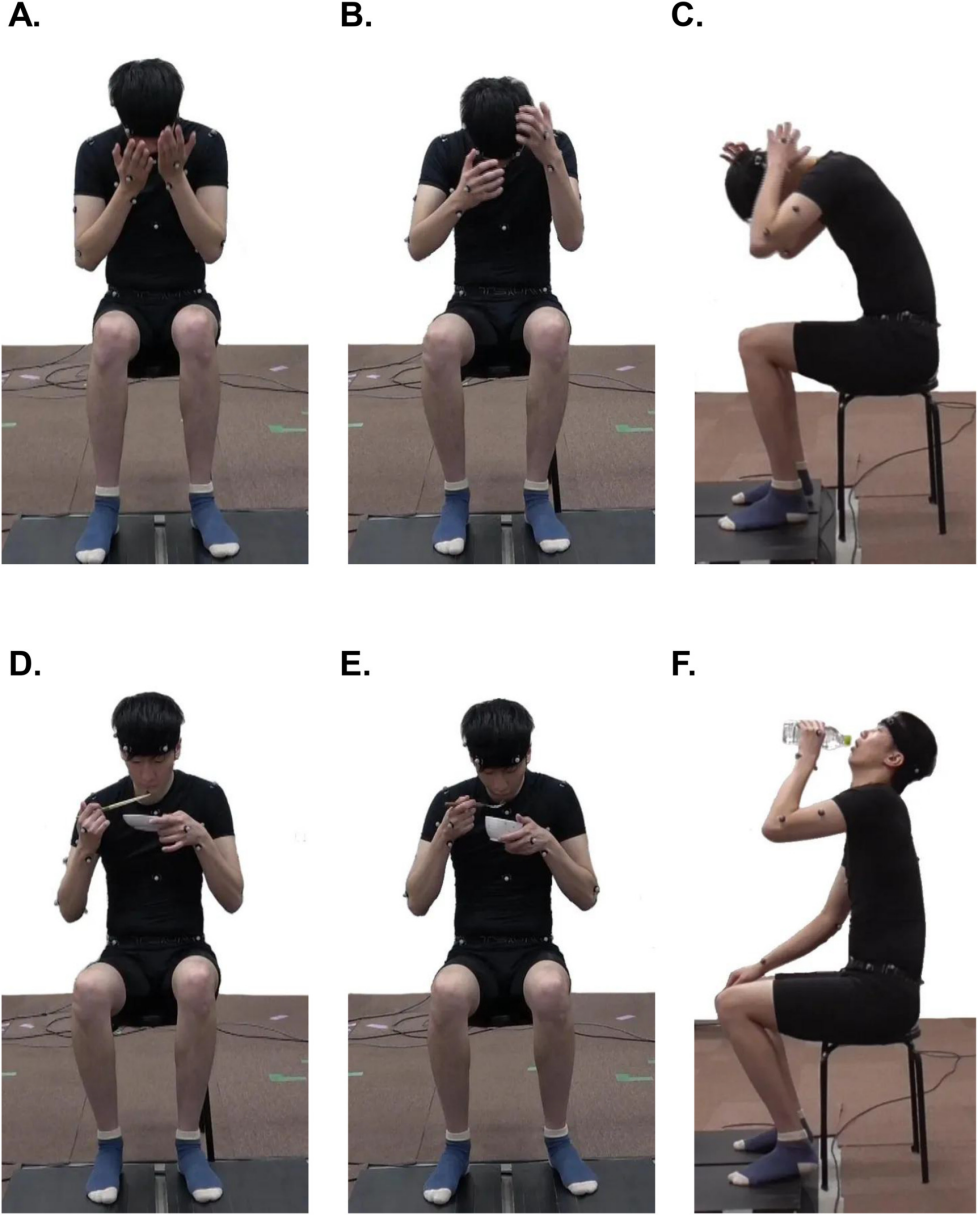
Six ADLs. **(A)** Fwashing, **(B)** washing hair at the top of the head, **(C)** washing hair at the back of the head, **(D)** eating with chopsticks, **(E)** eating with a spoon, **(F)** drinking bottled water.

The following six ADLs were investigated in this study: face washing, hair washing (at the top and back), eating with chopsticks or a spoon, and drinking bottled water. Participants were seated on a backless chair, with the chair height adjusted so that both feet rested flat on the floor. They were instructed to perform the six ADL tasks naturally, without receiving any specific or special instructions. For face washing, participants were asked to simulate face washing with approximately three strokes (Figure 1A). For hair washing, participants were asked to simulate hair washing on the top/at the back of the head with approximately three strokes (Figures 1B,C). For eating with chopsticks or a spoon, participants were asked to simulate eating pseudo food on a plate with chopsticks (Figure 1D) and in a bowl with a spoon (Figure 1E). For the eating task, participants were instructed to hold the chopsticks or spoon with their right hand and the bowl or plate with their left hand. For the drinking task, participants were asked to simulate drinking bottled water (500 ml) (Figure 1F). Participants were instructed to hold the bottle of water with their left hand and perform a simulated drinking motion.

#### 2.2.2 Data collection

**Figure 2:**
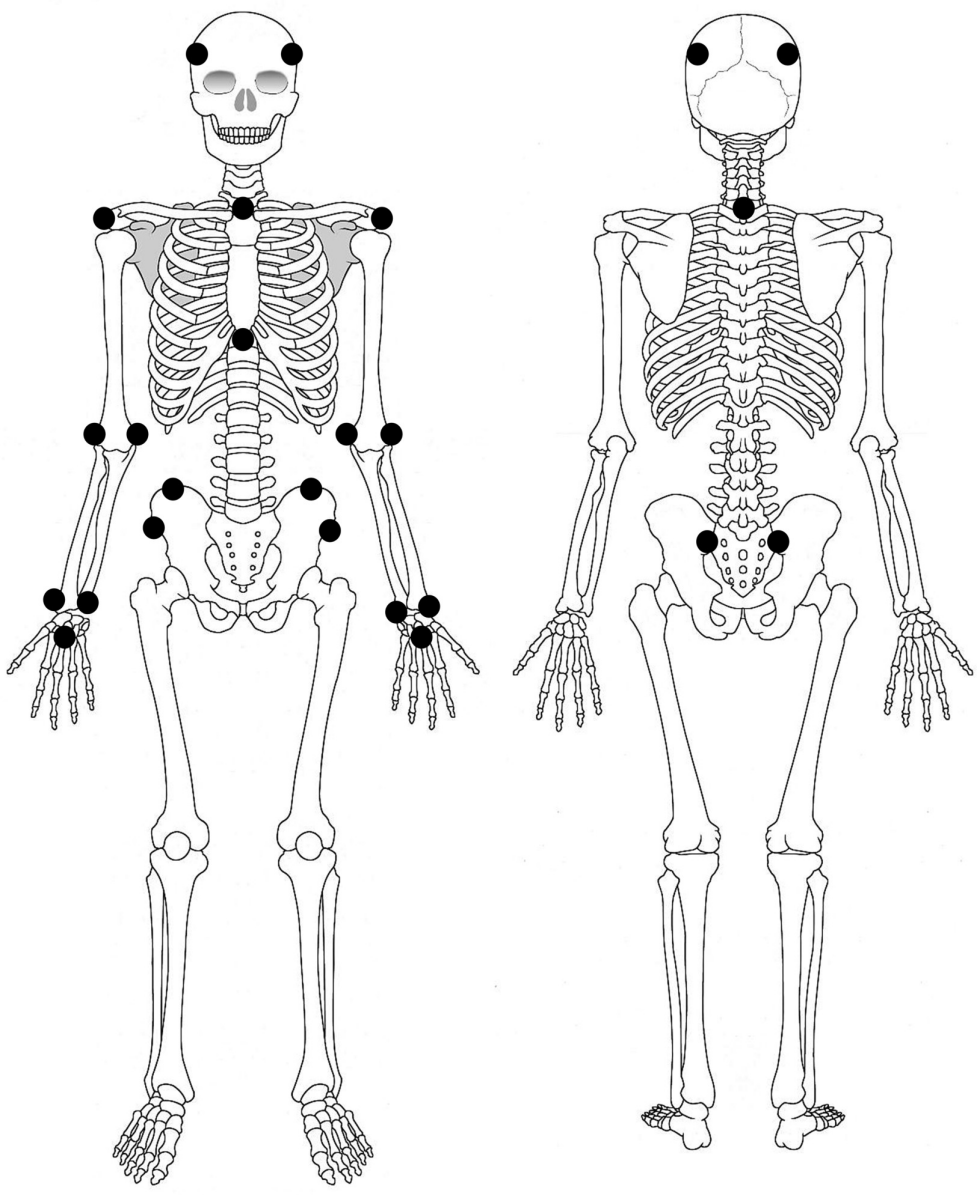
Marker arrangement of the three-dimensional motion analysis system. The positions of the retroreflective markers are indicated by black circles.

Data were collected using a marker-based motion capture system (Cortex version 5.0.1, Motion Analysis Corporation, Santa Rosa, CA, USA) with seven Hawk cameras (Motion Analysis Corporation). The sampling rate was set to 120 Hz. Marker placements were based on previous studies (28, 29). Retroreflective markers were placed on the head (right/left anterior, right/left posterior), the spinous process of the seventh cervical (C7) and eighth thoracic vertebra, suprasternal notch, xiphoid process, acromion, medial/lateral epicondyles of the humerus, radial processes, ulnar processes, the head of the third metacarpal, anterior superior iliac spines, posterior superior iliac spines, and iliac crest (Figure 2). The xiphoid process marker was attached to taped clothing to prevent lifting. First, a standardized static trial was recorded. Then, participants performed each ADL task while sitting. Participants were instructed to begin and end all tasks with their hands on their knees. Each task is described in the subheading “activity of daily living tasks” above. Participants performed each ADL task five times at a self-selected pace, with the first and second trials designated as practice to allow familiarization with the task. The order of the ADL tasks was randomized for each participant.

### 2.3 Data analysis

Kinematic analysis was performed using Visual3D (version 6, C-Motion, Inc., Germantown, MD, USA). The marker coordinates were filtered using a fourth-order, zero-lag Butterworth lowpass filter with a cutoff frequency of 6 Hz. The thorax, humerus, and forearm coordinate systems were defined according to the recommendations of the International Society of Biomechanics (28). The hand coordinate system was defined according to the previous report by Rab et al. (29). Axes of each segment's coordinate were defined as follows: *X*-axis (medial/lateral), *Y*-axis (anterior/posterior), and *Z*-axis (superior/inferior). The *Z*-axis of the head was defined as the line from the midpoint of C7 and the suprasternal notch to the midpoint of four head markers. The *X*-axis was perpendicular to the *Z*-axis, and the direction was determined based on the midpoint of the right anterior and posterior head markers. The *Y*-axis was perpendicular to the *X*- and *Z*-axes. The shoulder angle was calculated as the humeral motion relative to the thorax with a *Z*-*Y*-*Z* sequence. Thus, the first rotation corresponds to the plane of elevation, with 0° representing elevation in the frontal plane and 90° representing elevation in the sagittal plane. The second rotation represents the angle of elevation, while the third rotation corresponds to the axial internal/external rotation. Elbow flexion/extension and forearm pronation/supination were calculated as forearm motion relative to the humerus with an *X*-*Y*-*Z* sequence (flexion/extension, varus/valgus, and then pronation/supination). Wrist flexion/extension was calculated as hand motion relative to the forearm with an *X*-*Y*-*Z* sequence (flexion/extension is the first). Neck flexion/extension was calculated as head motion relative to the thorax with the *X*-*Y*-*Z* sequence. Thorax flexion was calculated as the thorax motion relative to the laboratory coordinate with *X*-*Y*-*Z* sequence.

Positive values indicated shoulder elevation, shoulder external rotation, elbow flexion, forearm pronation, wrist palmar flexion, trunk flexion, and neck flexion. Negative values indicated shoulder extension, shoulder internal rotation, elbow extension, forearm supination, wrist dorsiflexion, trunk extension, and neck extension.

The analyzed period was defined as the upward and downward velocity of the wrist joint (midpoint between the radial and ulnar processes). Movement onset was defined as the point when the upward velocity exceeded 20% of the peak velocity, while the offset was defined as the point when the downward velocity fell below 20% of the peak velocity. The peak joint angles were extracted from this period. Additionally, for each kinematics, the shoulder plane of elevation and rotation at peak elevation was derived. The kinematic joint angle waveforms were normalized to 101 data points (0%–100% of the movement cycle), and mean curves with 95% confidence intervals (CIs) were calculated to represent the motion patterns of each task. For each ADL task, the average value of the data obtained from the third, fourth, and fifth trials was used.

### 2.4 Statistical analysis

Descriptive statistics [mean, standard deviation (SD), and 95% CI] were calculated for the minimum and maximum joint angles across all tasks. For each task, the following joint movements were selected as the primary outcome variables: shoulder elevation, elevation plane during peak shoulder elevation, shoulder internal/external rotation, elbow flexion, forearm pronation/supination, wrist palmar/dorsiflexion, trunk flexion, and neck flexion. Paired t-tests were conducted to compare peak joint angles between subtask variants within individual ADLs. Specifically, comparisons were made between top-of-the-head vs. back-of-the-head hair washing, as well as between using chopsticks vs. a spoon, and between using a bowl vs. a dish during eating tasks. For all tasks, the right upper limb was the primary focus of the analysis. In addition, the ROM of the left upper limb was analyzed during tasks of drinking bottled water and grasping a dish or bowl as part of eating movements. To standardize the mean differences between the two groups, Cohen's d was calculated as a measure of effect size, with values ≥ 0.8 defined as indicating a “large effect size” (30). All statistical analyses were conducted using SPSS (version 30.0). Effects were considered significant at *p* < 0.05.

## 3 Results

### 3.1 Face washing

**Figure 3:**
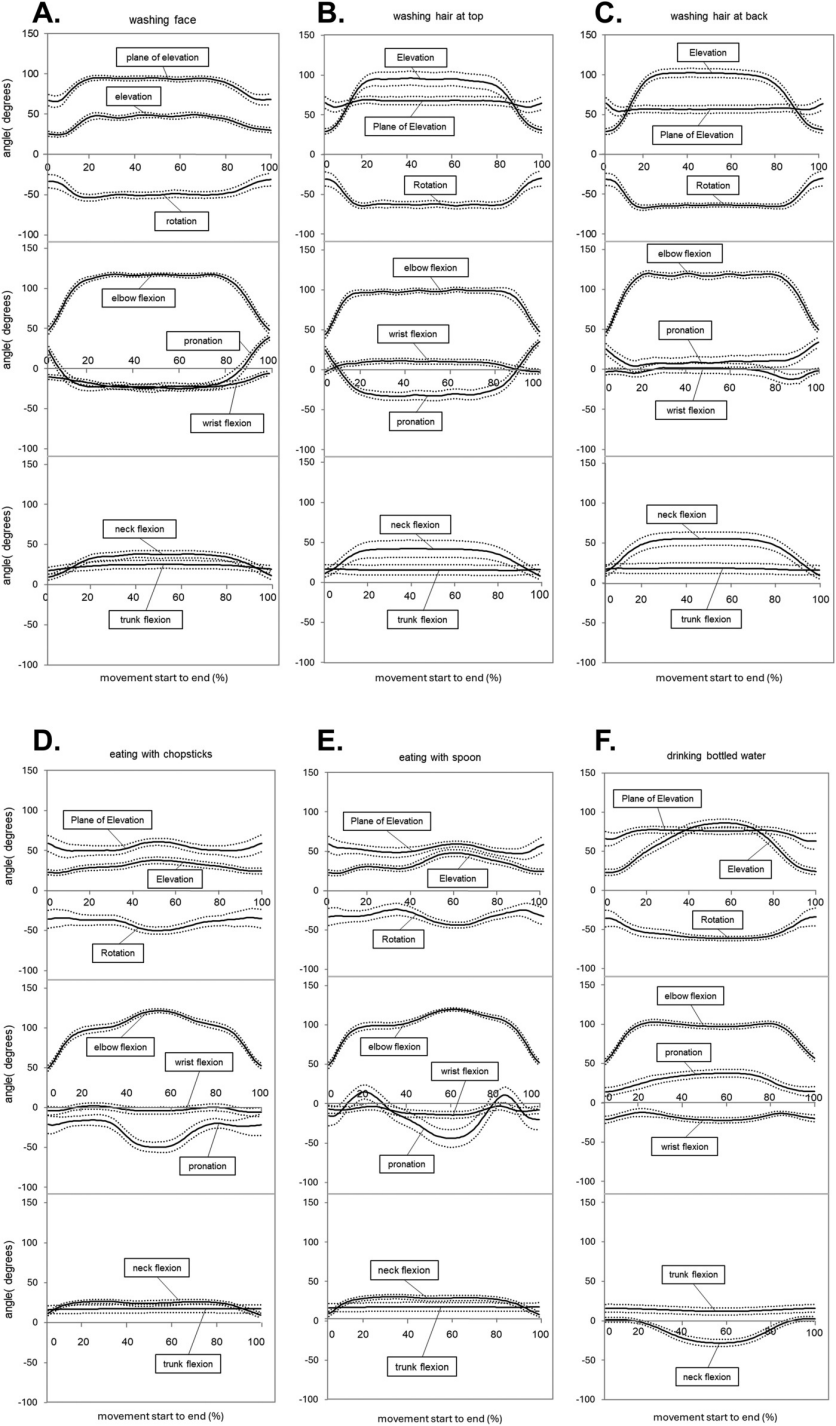
Time-normalized data for each task. The external rotation is a positive axial rotation, while the internal rotation is a negative axial rotation. Forearm pronation is a positive axial rotation, and forearm supination is a negative axial rotation. Flexion is positive, and extension is negative. **(A)** Face washing, **(B)** washing hair at the top of the head, **(C)** washing hair at the back of the head, **(D)** eating with chopsticks, **(E)** eating with a spoon, **(F)** drinking bottled water. **(A–E)** Represent the time-normalized data of the right upper limb, and **(F)** represents that of the left upper limb.

The normalized data for face washing were shown in Figure 3A. The maximum shoulder joint elevation was 54.9° ± 8.5°, and the plane of elevation nearly aligned with the sagittal plane (95.7° ± 8.7°) (Table 1). During elevation, the shoulder externally rotated to −59.7° ± 10.3°. The maximum elbow flexion reached 122.3° ± 5.2°, accompanied by −30.8° ± 15.1° of forearm supination (Table 2). The wrist joint extended to −28.7° ± 8.5°. The neck flexed from 6.0° ± 10.3° to 41.0° ± 12.1°, with minimal change in the trunk flexion angle (Table 3).

**Table 1:** Maximal, minimal, and standard deviation of the angle of shoulder motion.

ADL task	Laterality	Shoulder elevation		Plane of elevation	Rotation	
		Max (SD)	Min (SD)	At peak elevation (SD)	Max (SD)	Min (SD)
Face washing	Right	54.9 (8.5)	22.4 (7.0)	95.7 (8.7)	−24.5 (21.1)	−59.7 (10.3)
	Left	54.6 (9.0)	20.4 (7.9)	95.5 (9.9)	−24.1 (22.3)	−62.7 (10.9)
Washing hair at the top	Right	99.4 (23.3)	28.1 (9.4)	68.9 (14.6)^a^	−22.9 (26.3)	−71.1 (9.0)
	Left	97.9 (23.8)	26.3 (10.5)	69.2 (14.3)	−22.7 (32.0)	−73.0 (9.6)
Washing hair at the back	Right	105.0 (14.6)	27.3 (10.1)	56.9 (13.1)	−23.9 (25.4)	−72.3 (7.0)
	Left	102.7 (15.4)	26.7 (11.0)	57.6 (12.7)	−23.7 (30.7)	−74.2 (7.6)
Eating with chopsticks	Chopsticks	39.2 (10.9)	20.6 (8.7)	62.5 (12.8)^b^	−21.2 (26.6)	−56.4 (13.7)
	Dish	24.5 (10.3)	16.1 (8.1)	58.1 (30.1)	−8.5 (35.4)	−44.9 (20.9)
Eating with a spoon	Spoon	49.7 (11.1)^c^	19.9 (7.6)	61.0 (10.9)	−9.8 (26.4)	−51.8 (13.7)
	Bowl	27.4 (9.1)^d^	15.6 (6.8)	58.9 (28.4)	−8.8 (30.6)	−44.0 (18.2)
Drinking bottled water	Left	87.3 (12.8)	21.2 (10.5)	75.9 (11.0)	−25.9 (29.9)	−64.2 (9.0)

The external rotation is a positive axial rotation, while the internal rotation is a negative axial rotation. SD, standard deviation

^a^ Washing hair at the top > back;

^b^ Eating with chopsticks > a spoon;

^c^ Eating with a spoon > chopsticks;

^d^ Holding a bowl > a dish.

**Table 2:** Maximal, minimal, and standard deviation of the angle of the elbow, forearm, and wrist motion.

ADL task	Laterality	Elbow flexion		Forearm pronation		Wrist flexion	
		Max (SD)	Min (SD)	Max (SD)	Min (SD)	Max (SD)	Min (SD)
Face washing	Right	122.3 (5.2)	45.1 (13.5)	39.2 (9.4)	−30.8 (15.1)	−2.3 (9.1)	−28.7 (8.5)
	Left	131.6 (5.1)	50.5 (14.4)	41.8 (9.6)	−30.1 (15.0)	−2.3 (8.7)	−31.5 (9.8)
Washing hair at the top	Right	107.8 (10.4)^b^	42.4 (12.6)	36.3 (9.2)	−38.9 (13.8)^a^	15.8 (7.2)^a^	−9.1 (10.0)
	Left	117.3 (11.4)	47.4 (13.7)	38.2 (8.0)	−38.2 (11.9)	14.7 (8.7)	−11.9 (10.5)
Washing hair at the back	Right	127.9 (5.7)	44.6 (11.5)	36.2 (16.9)	−3.3 (17.1)	10.5 (10.7)	−20.9 (18.0)
	Left	136.4 (5.8)	48.9 (12.9)	38.5 (14.7)	−4.2 (15.8)	8.1 (8.4)	−22.0 (15.7)
Eating with chopsticks	Chopsticks	122.7 (6.0)	48.6 (9.4)	0.7 (26.1)	−54.1 (16.9)	8.5 (10.3)	−13.7 (12.5)^c^
	Dish	123.4 (8.3)	55.7 (8.9)	−9.4 (14.9)	−29.2 (16.8)	8.5 (9.5)	−10.6 (8.5)
Eating with a spoon	Spoon	121.4 (4.2)	48.2 (10.3)	27.1 (24.6)^d^	−49.0 (28.6)	7.4 (11.6)	−21.8 (11.0)
	Bowl	128.5 (7.6)^e^	53.3 (11.4)	0.7 (17.4)^e^	−37.6 (17.8)^e^	11.2 (10.4)^e^	−12.8 (8.5)^e^
Drinking bottled water	Left	106.5 (8.8)	51.7 (10.0)	40.9 (12.3)	5.2 (17.0)	−4.8 (13.5)	−29.4 (8.9)

Forearm pronation is a positive axial rotation, and forearm supination is a negative axial rotation. Wrist flexion is positive, and wrist extension is negative. SD, standard deviation.

^a^ Washing hair at the top > back;

^b^ Washing hair at the back > top;

^c^ Eating with chopsticks > a spoon;

^d^ Eating with a spoon > chopsticks;

^e^ Holding a bowl > a dish.

**Table 3:** Maximal, minimal, and standard deviation of the angle of the trunk and neck.

ADL task	Trunk flexion		Neck flexion	
	Max (SD)	Min (SD)	Max (SD)	Min (SD)
Face washing	26.7 (13.7)	16.2 (12.4)	41.0 (12.1)	6.0 (10.3)
Washing hair at the top	19.7 (16.2)^a^	12.2 (15.7)	46.6 (23.1)^a^	3.1 (14.9)
Washing hair at the back	22.5 (16.4)	12.8 (15.7)	58.1 (19.8)	6.5 (13.3)
Eating with chopsticks	18.8 (14.7)	15.7 (13.7)	30.1 (6.4)	7.7 (8.7)^b^
Eating with a spoon	19.1 (15.1)	15.5 (14.3)	33.6 (7.6)^c^	5.2 (8.3)
Drinking bottled water	16.9 (13.5)	11.4 (12.5)	6.9 (7.5)	−29.3 (11.6)

Flexion is positive, and extension is negative. SD, standard deviation.

^a^ Washing hair at the back > top;

^b^ Eating with chopsticks > a spoon;

^c^ Eating with a spoon > chopsticks.

### 3.2 Washing hair on the top

The normalized data for top-of-the-head hair washing are shown in Figure 3B. The shoulder elevation reached 99.4° ± 23.3°, with an elevation plane angle of 68.9° ± 14.6° and external rotation of −71.1° ± 9.0° (Table 1). The elbow flexed to 107.8° ± 10.4°, and the forearm supinated to −38.9° ± 13.8° (Table 2). The wrist joint flexed to 15.8° ± 7.2° (Table 2). The neck flexed from 3.1° ± 14.9° to 46.6° ± 23.1°, while the trunk flexion angle showed relatively small changes (Table 3).

### 3.3 Washing hair at the back

The normalized data for back-of-the-head hair washing are shown in Figure 3C. The maximum shoulder elevation and external rotational angle were 105.0° ± 14.6° and −72.3° ± 7.0°, respectively, and both were the greatest among the six tasks tested (Table 1). The plane of elevation was 56.9° ± 13.1°. The elbow flexion angle reached 127.9° ± 5.7°, with −3.3° ± 17.1° of supination (Table 2). The neck flexion angle was the greatest among the six ADL tasks, reaching 58.1° ± 19.8°. The trunk remained in a slightly flexed position (Table 3).

### 3.4 Eating with chopsticks

The normalized data for eating with chopsticks are shown in Figure 3D. The maximum shoulder elevation was 39.2° ± 10.9° for the chopstick side and 24.5° ± 10.3° for the dish side (Table 1). The elevation plane angle was similar for the chopstick and dish sides (Table 1). The shoulder was externally rotated for both sides. The maximum elbow flexion reached 122.7 ± 6.0 and 123.4° ± 8.3° for the chopstick and dish sides, respectively. The forearm was supinated at −54.1° ± 16.9° and −29.2° ± 16.8° for the chopstick and dish sides, respectively. The wrist extended to −13.7° ± 12.5° and −10.6° ± 8.5° for the chopstick and dish sides, respectively (Table 2). The neck flexed slightly, while the trunk flexion showed minimum changes (Table 3).

### 3.5 Eating with a spoon

The normalized data for eating with a spoon are shown in Figure 3E. The maximum shoulder elevation was 49.7° ± 11.1° for the spoon side, which was slightly larger than that of eating with chopsticks (Table 1). The elbow flexion angle was 121.4° ± 4.2°, with small variability among participants, while the forearm supination angle was 49.0° ± 28.6°, showing large variability (Table 2). The wrist joint remained in an approximately intermediate position. The trunk and neck were in a mildly flexed position (Table 3).

### 3.6 Drinking bottled water

The normalized data for drinking bottled water are shown in Figure 3F. The maximum shoulder elevation was approximately 87.3° ± 28.6°, and the plane of elevation was 75.9° ± 11.0° (Table 1). The forearm and wrist were fixed in a pronation and extension position entirely (Table 2). Neck flexion was the only negative value observed among the six ADLs, reaching −29.4° ± 8.9° (Table 3).

### 3.7 Comparison of subtask variants

#### 3.7.1 Hair washing: top vs. back

The elevation plane was greater when washing the top than that with the back [*t* (30) = 6.70, *p* < 0.001, *d* = 1.20, 95% CI (8.32, 15.63)] (Table 1). The forearm supination and wrist flexion angle were also greater for top-of-the-head than those of back-of-the-head hair washing [forearm supination: *t* (30) = –10.96, *p* < 0.001, *d* = 1.97, 95% CI (–42.19, −28.94); wrist flexion: *t* (30) = 2.92, *p* = 0.0073, d = 0.53, 95% CI (1.58, 8.91)] (Table 2). In contrast, the maximum elbow flexion was significantly greater when washing the back than when washing the top [*t* (30) = −14.36, *p* < 0.001, *d* = −2.58, 95% CI (−23.15, −17.38)] (Table 2). For the trunk and neck, the flexion angle was significantly greater when washing the back than when washing the top [trunk: *t* (30) = –2.15, *p* = 0.04, *d* = –0.39, 95% CI (–5.37, −0.14), neck: *t* (30) = –4.49, *p* < 0.001, *d* = –0.81, 95% CI (–16.70, −6.26)] (Table 3).

#### 3.7.2 Eating: chopsticks vs. spoon manipulation

The elevation plane was greater when eating with chopsticks than when eating with a spoon [*t* (30) = 5.73, *p* < 0.001, *d* = 1.02, 95% CI (5.51, 11.62)] (Table 1). Wrist and neck extensions were also greater when eating with chopsticks than that when eating with a spoon [wrist extension: *t* (30) = 3.05, *p* = 0.005, *d* = 0.55, 95% CI (2.69, 13.61) (Table 2), neck extension: *t* (30) = 2.10, *p* = 0.04, *d* = 0.38, 95% CI (0.064, 4.86)] (Table 3). In contrast, the maximum shoulder elevation when eating with a spoon was significantly greater than that when eating with chopsticks [*t* (30) = –8.81, *p* < 0.001, *d* = –1.58, 95% CI (–12.94, −8.07)] (Table 1). Additionally, forearm rotation and neck flexion were greater when eating with a spoon than that when eating with chopsticks [*t* (30) = –4.67, *p* < 0.001, d = –0.84, 95% CI (–38.09, −14.90), neck: *t* (30) = –3.97, *p* < 0.001, d = –0.71, 95% CI (–5.37, −1.72)] (Tables 2, 3).

#### 3.7.3 Eating: dish vs. bowl grasping

Shoulder elevation, elbow flexion, forearm pronation, supination, wrist flexion, and extension were significantly greater during bowl grasping than that during plate grasping (shoulder elevation: *t* (30) = –3.29, *p* = 0.003, *d* = –0.59, 95% CI [–4.67, −1.11], elbow flexion: (*t* (30) = –5.13, *p* < 0.001, *d* = –0.92, 95% CI [–7.11, −3.06], forearm pronation: *t* (30) = –4.74, *p* < 0.001, *d* = –0.85, 95% CI [–14.44, −5.74], forearm supination: (*t* (30) = 4.66, *p* < 0.001, *d* = 0.84, 95% CI [4.71, 12.07], wrist flexion: *t* (30) = –2.34, *p* = 0.03, *d* = –0.42, 95% CI [–5.11, −0.35]), wrist extension: *t* (30) = 2.28, *p* = 0.042, *d* = 0.38, 95% CI [0.08, 4.49] (Tables 1, 2).

## 4 Discussion

In this study, we focused on ADL tasks commonly performed in a seated position in Japan, such as face washing, hair washing, and eating, taking into account the country's cultural background. We analyzed the kinematic characteristics of each task and further compared movement patterns when the same ADL was performed using different methods.

### 4.1 Face washing

The face-washing motion required relatively large elbow joint flexion and wrist joint extension to raise both hands to the face. According to a 3D motion analysis of healthy adult participants, the wrist extension angle during face washing reached approximately 30°, indicating the importance of wrist joint extension when placing the hands on the face (7). Previous studies (7, 25) reported shoulder elevation angles of 50° ± 7° and 44° ± 10° during face washing. Our study showed greater shoulder elevation angles (54.9° ± 8.5°) than those previously reported. In contrast, the elbow flexion angle in the present study was slightly lower (122.3° ± 5.2°) than the 140° ± 5° and 128° ± 6° reported by Henmi et al. (25) and Aizawa et al. (7), respectively. The neck flexion angle in the present study was 41.0° ± 12.1°, which was greater than that reported by Henmi et al. (25) (16° ± 7°). Furthermore, the trunk flexion showed the greatest ROM among the six ADLs analyzed in the present study. Differences were observed in the shoulder and elbow joint angles compared with those in previous studies, and neck and trunk flexion were relatively large, suggesting differences in movement strategies. The previous study did not specify the posture (standing or sitting) in which the face-washing motion was performed; however, our data in the present study were collected when participants were in a sitting position. Differences in body position may have contributed to the differences in the kinematics between the present and previous studies. In the sitting position, part of the elbow and shoulder joint ROM may be compensated for by the neck and trunk flexion.

### 4.2 Washing hair: top vs. back

Hair washing required elevation of the upper limbs to a higher position than that of face washing, especially when washing the top and back of the head, and required a wider ROM of the shoulder joints. Previous studies on healthy subjects have shown that a minimum shoulder elevation of approximately 73° was required for hair combing and washing, with the angle of elevation generally ranging from 0° to 100° (8). A previous study (24) reported a shoulder internal rotation angle of 96.7° ± 19.5° in the hair-washing motion performed while standing. However, in this study, the internal rotation of the shoulder joint was approximately 71° and 74° for top-of-the-head and back-of-the-head hair washing, respectively, suggesting that the shoulder rotation angle may be slightly smaller in a seated operating environment than that in a standing environment. In contrast, for the elbow joint, approximately 110°–120° of flexion was required to reach the hair, and the minimum elbow flexion angle was 112°, as reported in a previous study (8). In this study, an elbow joint flexion of 107.8° ± 10.4° and 127.9° ± 5.7° was observed when washing the hair on the top and at the back of the head, respectively, with a trend toward greater flexion angles, especially when washing the hair at the back of the head. This suggests that the elbow joint might have been subjected to an increased ROM requirement in actual hair-washing situations, which involve rubbing the top and back of the head with the fingers.

### 4.3 Comparison: washing hair on the top vs. the back

In this study, we compared the functional ROM during hair-washing at the top vs. the back and found that, despite the nominally identical task, the movement strategies employed differed markedly. When washing the vertex, participants exhibited shoulder elevation following a trajectory closer to the sagittal plane, accompanied by forearm supination and wrist palmar flexion to maintain the palm facing upward, enabling a smooth reach to the crown. In contrast, washing the occiput was characterized primarily by deep elbow flexion and pronounced cervical flexion to reduce the distance between the hand and the back of the head, accompanied by slight trunk flexion to stabilize the head position. These observations align with previously reported characteristics of combing movements at the occipital region (7) and substantiate that the required ROM varies by target region. Accordingly, training and assessment protocols for independent hair washing should emphasize shoulder elevation control and the acquisition of forearm supination and wrist palmar flexion when targeting the vertex, whereas interventions focusing on the occiput should prioritize achieving sufficient elbow flexion and cervical/trunk mobility.

### 4.4 Eating with chopsticks or a spoon

In addition to the skillful finger and hand joint movements associated with chopstick and spoon manipulation, the eating motion requires upper limb ROM to transport food to the mouth. A study involving young, healthy individuals reported that the elbow joint reached a maximum flexion of approximately 129°, and the wrist joint reached a maximum extension of approximately 32° during eating motions (31). Magermans et al. (8) suggested that the most important joint for eating movements required elbow flexion of at least 117°, consistent with the findings of our study (approximately 121°). A previous study where chopstick and spoon manipulation were compared reported significant differences in shoulder joint flexion/shoulder abduction, elbow joint flexion, forearm rotation, and wrist joint extension/radial flexion angles (26). Generally/, chopstick manipulation requires precise hand control, while spoon manipulation requires shoulder and elbow angles suitable for the scooping motion. In this present study, shoulder flexion, forearm pronation, and wrist extension were greater during spoon manipulation than during chopstick manipulation, and neck flexion was also greater during spoon manipulation than during chopstick manipulation. These differences may reflect both the position of the tableware and the holding method during eating situations (26). In the Japanese bowl-eating culture, it is customary to elevate the upper limb when holding the bowl in the hand. Compared with the findings of the present study, the shoulder joint elevation plane angle and elbow joint flexion angle were significantly greater when holding the bowl than when holding the dish, suggesting that the movement of holding the dish close to the mouth increases the upper limb ROM demand.

### 4.5 Drinking bottled water

The substantial recruitment of shoulder elevation and elbow flexion observed in the drinking motion in this study was consistent with that of a previous study (7). However, the wrist extension angles recorded here (approximately 30°) exceeded the previously reported values of 15° ± 13° (7), suggesting that participants manipulated the PET bottle's tilt through active wrist extension. Furthermore, while only the present study reported negative neck angles (approximately 29° of extension), this degree of extension was smaller than the 43°–60° range reported in earlier studies (32). This indicates that bottle orientation when using a PET bottle can be controlled via wrist extension and forearm pronation, thereby reducing the need for large cervical extension.

These results suggested that drinking from a PET bottle involved a two-stage movement strategy: (a) reaching via shoulder elevation and elbow flexion, followed by (b) bottle-tilt adjustment through wrist extension and forearm pronation. This reduced the biomechanical demand on cervical extension.

The functional ROM identified for each ADL task in this study provides valuable clinical guidelines for rehabilitation. Assessment and training for face-washing should emphasize elbow flexion and wrist extension. Hair-washing (top-of-the-head) assessments should focus on control of the plane of shoulder elevation and acquisition of forearm supination and wrist palmar flexion, whereas back-of-the-head hair-washing requires targeted development of elbow flexion and cervical/trunk flexion. In chopstick use, wrist extension and fine cervical adjustments are paramount, while spoon use requires shoulder elevation with internal/external rotation and forearm pronation. Furthermore, utensil stabilization, especially with a bowl, relies critically on adequate elevation and flexion range. The drinking motion should be conceptualized as a two-stage strategy: reaching via shoulder elevation and elbow flexion, followed by instrument-tilt adjustment through wrist extension and forearm pronation. These insights support the design of rehabilitation programs that set joint-range goals tailored to the specific task and equipment, thereby enhancing ADL independence and movement quality.

This study has some limitations. First, only young, healthy adults were included. Therefore, the applicability of the findings to clinical populations, for whom ROM normative values are critical (such as older adults, post-stroke survivors, or patients with orthopedic impairments), remains unclear. Caution is warranted when applying these reference values directly to these patient groups. Second, since the ADL tasks were simulated under naturalistic conditions without a standardized execution framework, there was greater inter-individual variability than in many previous studies. Third, the use of a “simulated” ADL format does not fully reproduce real-life performance, so movement patterns may differ from those in everyday contexts. Fourth, no statistical tests for bilateral asymmetry were conducted. While tasks such as face- and hair-washing involve symmetric, bilateral movements, laterality analyses should be included in future studies conducted to address ADLs that require asymmetric limb actions, such as dressing. Fifth, the study design required participants to perform ADLs in a seated position, which limits the generalizability of the results to standing or ambulatory contexts. Sixth, instructing participants to hold dishes with their non-dominant hand reflects Japanese cultural practices, limiting the applicability of the findings to other cultural settings. Future research should be conducted to address these limitations by including clinical populations, implementing standardized movement protocols, testing for laterality effects, measuring ADLs in various postures, and validating findings in larger samples encompassing older adults and patient groups.

In this study, we quantified the functional ROM of the upper limbs, trunk, and cervical spine required for four seated ADL tasks—face washing, hair washing, eating, and drinking—in young, healthy adults performing movements characteristic of Japanese culture. We also compared movement patterns for the same ADLs executed by different methods: top-of-the-head vs. back-of-the-head hair washing, chopstick vs. spoon eating, and plate vs. bowl manipulation.

Our findings suggested that face washing required approximately 122° of elbow flexion, 29° of wrist extension, and 41° of cervical flexion. For top-of-the-head hair washing, control of the shoulder-elevation plane combined with forearm supination and wrist flexion was the dominant strategy, whereas back-of-the-head hair washing relied primarily on deep elbow flexion and flexion of the cervical spine and trunk. For eating, chopstick use involved modest wrist extension and fine cervical adjustments, whereas spoon use required greater shoulder elevation, forearm pronation, and cervical flexion; holding a bowl, as opposed to holding a plate, further increased the ROM demands of shoulder elevation and elbow flexion. For the drinking task, a two-stage strategy—reaching via shoulder elevation and elbow flexion, followed by bottle-tilt adjustment through wrist extension and forearm pronation—appeared to reduce the biomechanical demand on cervical extension.

Across all ADL tasks, statistically significant differences in joint kinematics were accompanied by moderate to large effect sizes (*d* = 0.5–2.0), many of which exceeded typical thresholds for clinically meaningful change (33). These findings underscore that the detected variations are not only statistically valid but also practically significant in guiding individualized rehabilitation strategies. These quantitative ROM benchmarks provide clinically relevant targets for rehabilitation program design and outcome measurement, enabling the specification of joint-range goals tailored to each task and its implementation. Future work should extend these guidelines to older adults and clinical populations, incorporate standing and ambulation contexts, examine bilateral asymmetries, and account for cultural differences to establish more universally applicable and clinically robust functional ROM standards.

## Data Availability

The raw data supporting the conclusions of this article will be made available by the authors, without undue reservation.
